# A pipeline‐friendly software tool for genome diagnostics to prioritize genes by matching patient symptoms to literature

**DOI:** 10.1002/ggn2.10023

**Published:** 2020-08-10

**Authors:** K. Joeri van der Velde, Sander van den Hoek, Freerk van Dijk, Dennis Hendriksen, Cleo C. van Diemen, Lennart F. Johansson, Kristin M. Abbott, Patrick Deelen, Birgit Sikkema‐Raddatz, Morris A. Swertz

**Affiliations:** ^1^ Genomics Coordination Center University of Groningen and University Medical Center Groningen Groningen The Netherlands; ^2^ Department of Genetics University of Groningen and University Medical Center Groningen Groningen The Netherlands; ^3^ Prinses Maxima Center for Child Oncology Utrecht The Netherlands

**Keywords:** benchmark, command‐line, gene prioritization, genome diagnostics, next‐generation sequencing, patient symptoms, primary literature

## Abstract

Despite an explosive growth of next‐generation sequencing data, genome diagnostics only provides a molecular diagnosis to a minority of patients. Software tools that prioritize genes based on patient symptoms using known gene‐disease associations may complement variant filtering and interpretation to increase chances of success. However, many of these tools cannot be used in practice because they are embedded within variant prioritization algorithms, or exist as remote services that cannot be relied upon or are unacceptable because of legal/ethical barriers. In addition, many tools are not designed for command‐line usage, closed‐source, abandoned, or unavailable. We present Variant Interpretation using Biomedical literature Evidence (VIBE), a tool to prioritize disease genes based on Human Phenotype Ontology codes. VIBE is a locally installed executable that ensures operational availability and is built upon DisGeNET‐RDF, a comprehensive knowledge platform containing gene‐disease associations mostly from literature and variant‐disease associations mostly from curated source databases. VIBE's command‐line interface and output are designed for easy incorporation into bioinformatic pipelines that annotate and prioritize variants for further clinical interpretation. We evaluate VIBE in a benchmark based on 305 patient cases alongside seven other tools. Our results demonstrate that VIBE offers consistent performance with few cases missed, but we also find high complementarity among all tested tools. VIBE is a powerful, free, open source and locally installable solution for prioritizing genes based on patient symptoms. Project source code, documentation, benchmark and executables are available at https://github.com/molgenis/vibe.

## Graphical abstract

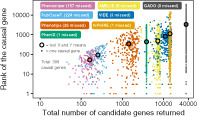

## 1 BACKGROUND

Next‐generation sequencing of the human genome enables clinical geneticists and medical researchers to establish molecular diagnoses for hereditary rare diseases.^1^, ^2^ However, despite explosive data growth^3^ and time‐consuming best efforts, chances of successfully detecting a causal variant are 40% at best.^4^, ^5^, ^6^, ^7^

A typical genome diagnostic analysis is performed by an automated reduction of millions of variants to a few dozen candidates for final interpretation by human experts. This reduction is accomplished by filtering variants based on genomic annotations such as allele frequency,^8^ inheritance mode,^9^ in silico pathogenicity estimates^10^ or previous classification.^11^

The resulting list of candidate variants can be further filtered or prioritized using the phenotype (ie, symptoms) of a patient. This is achieved by taking advantage of known associations between clinically relevant genes^12^, ^13^, ^14^ and clinical phenotypes, typically captured by Human Phenotype Ontology (HPO)^15^, ^16^ terms. These associations are stored in structured data sources^17^ and may also be extracted from clinical records^18^ or text mined from primary literature.^19^ Software tools have been developed that perform matching of patient symptoms to gene‐phenotype associations, followed by producing a list of prioritized genes based on how well they fit the phenotype of the patient.^20^, ^21^, ^22^, ^23^, ^24^, ^25^, ^26^ The list of prioritized genes allows experts to focus their attention on the most likely candidate variants first.

Despite many developments, few phenotype‐based gene prioritizers are suitable for routine use in automated molecular diagnostic pipelines. One key issue is the embedding of phenotype‐gene matching inside variant prioritization algorithms,^24^, ^27^ which means that the gene prioritization step is only applied to candidate SNVs (single nucleotide variants) and indels (small insertions and deletions). Additional molecular information coming from large structural variation,^28^, ^29^ comparative genomic hybridization, Sanger sequencing of poorly covered regions, cytogenetic observations, RNA‐sequencing^30^ and metabolomics^31^ cannot be taken into account, while diagnostic practice has shown that all available data must be analyzed in unison to achieve maximum yield.^32^ Combining many methods to analyze these different molecular data modalities, including gene and variant prioritizers, into a single monolithic application is not a sustainable solution. Therefore, VIBE focuses on providing candidate genes based on patient symptoms and can be used as an interchangeable module in composable pipelines for complex analyses.

Another key issue is not being able to install software locally. Tools that operate via a web‐interface or web‐API (Application Programming Interface)^24^, ^33^, ^34^, ^35^ would not work in a routine diagnostic setting, because they cannot depend on availability of external services and because of perceived legal or ethical barriers of sending patient details outside. Other blocking issues include tools not being designed for command‐line usage,^36^ source code or executables not being openly available,^37^, ^38^ and software being abandoned^39^ or no longer being available at all.^40^, ^41^, ^42^

We have developed VIBE (Variant Interpretation using Biomedical literature Evidence), an open‐source software tool that prioritizes disease genes that have been reported in literature, animal models and curated sources by considering patient symptoms. In contrast to the most comparable tools, this software is available as a stand‐alone command‐line executable which trivializes local integration into bioinformatic pipelines, allowing for use in routine genome diagnostics. By doing so, VIBE may save a significant amount of time by automating an otherwise labor‐intensive process, thereby speeding up diagnosis.

## 2 IMPLEMENTATION

VIBE (version 2.0) was programmed in Java 8,^43^ using DisGeNET^44^ as its main data source. DisGeNET is a discovery platform containing one of the largest publicly available collections of genes and variants associated to human diseases. It integrates data from expert curated repositories, GWAS (Genome‐Wide Association Study) catalogs, animal models and scientific literature.^45^ The value that VIBE adds to DisGeNET for use in genome diagnostics is that it (a) provides a quality open‐source command‐line executable, (b) semantically integrates DisGeNET with additional resources, (c) allows users to prioritize genes by HPO codes, and (d) runs offline to ensure availability and reproducibility.

The core data source of VIBE v2.0 is the RDF (Resource Description Framework) representation^46^ of DisGeNET r6.0. These data were supplemented with pda.ttl, phenotype.ttl, and void.ttl from DisGeNET r5.0. We also included SIO (Semanticscience Integrated Ontology, v1.43),^47^ used by DisGeNET to semantically harmonize its gene‐disease associations. Lastly, we incorporated Orphadata HOOM (The HPO‐ORDO Ontological Module) r1.3,^16^ which adds additional phenotype‐disease associations.

All these sources are combined into a TDB triple store, which is built using Apache Jena (v3.12.0).^48^ On this triple store, a SPARQL *construct* query is executed to obtain a minimized dataset in TTL format. The minimized TTL set is then used to build the final TDB triple store by Apache Jena. This database can be downloaded and used directly by VIBE. Alternatively, a shell script and detailed instructions are provided to build a custom database by the users themselves.

After data preparation, VIBE can be executed as a stand‐alone executable and works completely offline. Patient symptoms are accepted as the HPO codes from which optimized SPARQL queries are constructed to interrogate the triple store. Query output is internally parsed, processed and formatted for writing to an output file. VIBE comes with a unit test suite written in TestNG (v7.0.0)^49^ and is compiled using Apache Maven (v3.3.9).^50^

Availability and requirements:

Project name: VIBE
Project home page: https://github.com/molgenis/vibe
Operating system(s): Platform independent
Programming language: Java
Other requirements: Java 8 or higher
License: GNU Lesser General Public License v3.0
Any restrictions to use by nonacademics: None

### 2.1 Input parameters

The minimal set of VIBE command‐line input parameters consists of: *−t* pointing to the TDB triple store directory, *−o* denoting the output file location, and *− p* providing one or multiple HPO codes, for example, HP:0002996. Nonrequired, advanced options are: *−l* for genes‐only output, *−m* to set maximum ontology distance traversal, *−n* to select child or distance based ontology traversal, *−w* to supply an HPO OWL file when using the ‐m and ‐n options to increase phenotypic search space, *−v* for running in verbose mode, and finally, *−h* to print help.

### 2.2 Algorithm

Users provide one or multiple HPO codes as input search terms. If the *−w* option is used to supply an HPO OWL file along with *−m* > 0 and *−n* set to *distance*, all neighboring HPO terms at traversal distance *m* are added to the search terms. If *−n* set to *children*, only descendants of the input terms are considered here within the defined distance *−m*. VIBE first maps the HPO search terms to CUIs (concept unique identifiers) from UMLS (Unified Medical Language System^51^) using a SPARQL query. The query branches into three paths to retrieve CUIs of any Diseases, Disorders or Findings: (a) HPO to CUI matching according to UMLS Metathesaurus, (b) HPO to ORDO matching according to HOOM, (c) HPO to the gene‐diseases associations according to DisGeNET PDAs (Phenotype‐Disease Annotations). A union of resulting CUIs is then used to retrieve all matching GDAs (Gene‐Disease Associations). The GDAs are grouped by unique NCBI (National Center for Biotechnology Information) gene identifiers. The highest GDA score^52^ within each group determines the priority of the corresponding gene. The output is formed by listing all found genes in descending order of priority, accompanied by all GDA scores and PubMed identifiers of supporting literature grouped per CUI matched to that gene. All SPARQL queries and algorithms for data pre‐ and post‐processing can be found in the main VIBE repository.

### 2.3 Output file

The default output produced by VIBE is a tab‐delimited file containing three columns: (a) *gene* (NCBI), an NCBI gene identifier, (b) *highest GDA score* is the highest Gene‐Disease Association score from any of the associated diseases, disorders or findings, and lastly, (c) *diseases (UMLS) with sources per disease*, containing one or multiple associations represented by UMLS identifiers (eg, C0410538) plus GDA score and PubMed identifiers when available. Multiple associations are pipe‐separated. The genes‐only output contains only comma‐separated NCBI gene identifiers in descending order of relevance according to highest GDA score.

### 2.4 Patient benchmark

We constructed a benchmark using the reported symptoms and 308 causal genes from 305 rare disease patient cases, including three patients who received a dual molecular genetic diagnosis.^53^ Because these are published patient cases, their disease‐gene associations could have been included in DisGeNET, causing circular reasoning and therefore an unfair comparison. Consequently, we first made sure that this publication was not part of the DisGeNET data. The HPO terms from these cases were then matched to the HPO (release v2018‐03‐08) to obtain their corresponding codes. For details on processing the patient benchmark data, see Data 1 (Supporting Information). The resulting HPO codes were supplied to VIBE and seven other available tools that can prioritize genes based on phenotypes: Phenomizer,^38^ PhenoTips,^36^ hiPHIVE,^54^ PhenIX,^55^ PubCaseFinder,^35^ AMELIE,^34^ and GADO.^26^ The scope of these tools differs from known clinical genes (eg, Phenomizer), to genes mined from literature (eg, AMELIE), and gene expression‐based predictions for all coding and noncoding genes in GADO.

To benchmark PhenIX and hiPHIVE, we used the exomiser‐rest‐prioritizer module of the Exomiser open‐source code (release 12.1.0) to run a service that was able to prioritize genes based on HPO codes only, without the need to supply a VCF file. We used data version 1909 and default arguments. For GADO, we used the stand‐alone command‐line version 1.0.1 with prediction matrix hpo_predictions_sigOnly_spiked_01_02_‐2018. We accepted all automatically suggested alternative HPO terms in cases that the supplied HPO term could not be used. PhenoTips was benchmarked by running the “All‐in‐one package for OS X,” version 1.3.7. VIBE (version 2.0) was run at default settings without ontology traversal. The other tools were accessed via the web (AMELIE and Phenomizer during May/June 2018, PubCaseFinder in January 2020). Multiple queries were submitted in case input was restricted to a small set of genes to obtain a potential ranking for all genes. Python and R scripts were written to retrieve, merge, process and visualize the output gene lists from each assessed tool, and are available in the supplementary VIBE repository.

To find out how the tools would perform in a real‐life scenario, we also simulated the interpretation of a clinical exome. In this scenario, we suppose that a human expert is faced with 20 genes harboring candidate pathogenic variants of which one gene is causal. The expert uses a phenotype‐based gene prioritization tool to rank these 20 genes, followed by interpreting the variants starting from the most likely gene. To simulate this, we downloaded the CGD^12^ (Clinical Genomic Database, accessed 4 February 2020) to represent genes that could appear as candidates in a clinical exome analysis, and converted the gene names to NCBI identifiers (n = 3986). For each of the 308 causal genes from the patient cases, we selected 19 other pseudorandom genes from CGD and spiked in the causal gene. We then let each of the tools rank these 20 genes and retrieved the rank of the causal gene. If a gene could not be ranked because it was not present in the output of a tool, it was assigned a random rank positioned after the genes that were returned, because this is what would happen in practice. Suppose a tool returns a ranking for 15 of the 20 genes without the causal gene, then these 15 are investigated first, so the causal gene is later found anywhere between 16 and 20. To compensate for outliers, we stabilized the rank as the median of 25 permutations. Finally, we counted per tool the number of causal genes ranked first, second, third, and so on.

## 3 RESULTS

**FIGURE 1:**
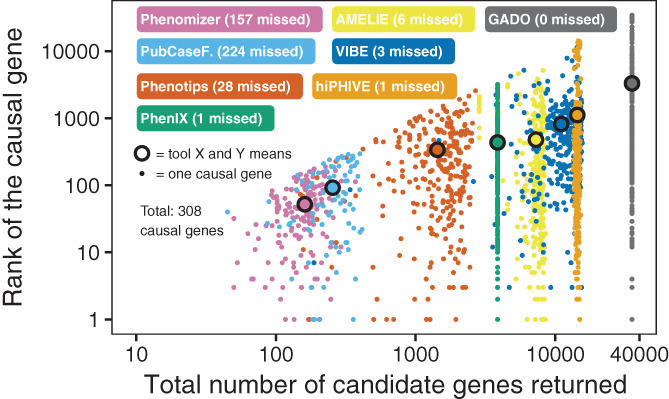
Gene prioritization tool output and causal gene rank for all patient cases. Each dot represents a patient case (ie, set of Human Phenotype Ontology codes) for which the causal gene was prioritized by one of eight benchmarked tools. Shown are the absolute ranks of the causal genes vs the total number of candidate genes returned by a tool. The colored labels indicate which dot belongs to which tool, as well as show the number of missed genes for each tool, where the causal gene was not present in the output gene list

To assess the behavior and performance of VIBE, we ran the benchmark described above. From each tool we obtained a list of prioritized genes for every patient case. Figure 1 shows the number of returned genes vs the rank of the causal gene if found within the output gene list. The number of missed genes for each tool, where the causal gene was not present in the output gene list, was added to the labels.

**FIGURE 2:**
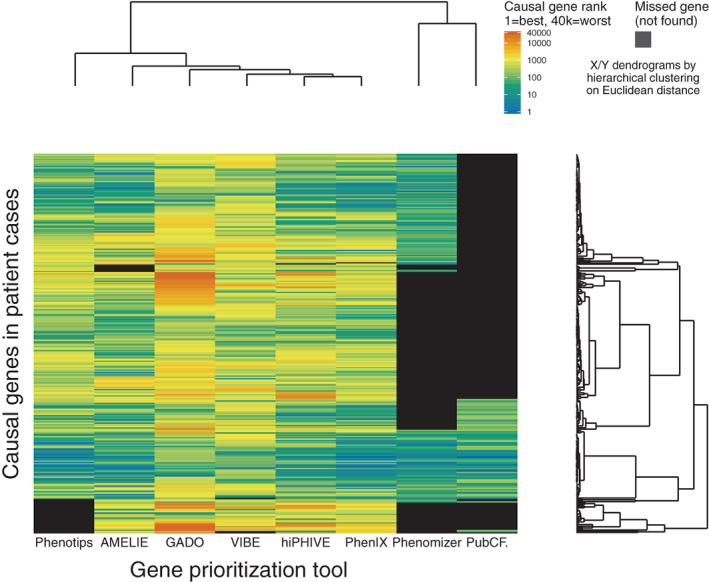
Heat map of case‐by‐case causal gene prioritization. Each bar represents a patient case (ie, set of Human Phenotype Ontology codes) for which the causal gene was prioritized by one of eight benchmarked tools. The color indicates the absolute causal gene rank within the output gene list, closer to one is better. Shown in black are causal genes that were not present in the output gene list of a tool. In total 308 bars are plotted because three of the 305 patient cases were affected by two disease causing genes

A heat map of the benchmark results is shown in Figure 2. For each patient case, we plotted the causal gene rank for all assessed tools. Causal genes that were not observed in the tool output are shown in black. Hierarchical clustering shows a degree of dissimilarity between all of the tested tools.

**FIGURE 3:**
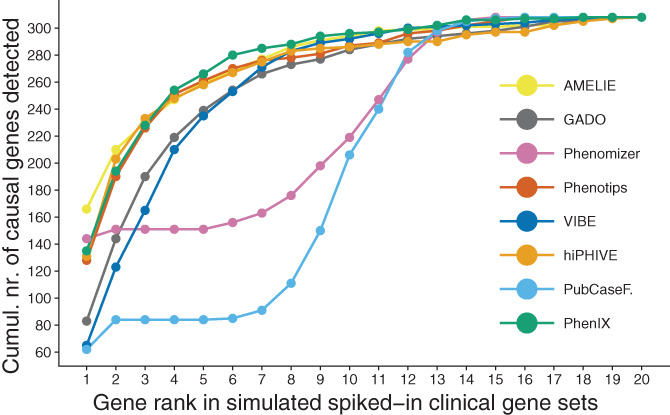
Results of the clinical exome interpretation simulation. Each dot represents the cumulative number of detected causal genes starting from rank 1 (best) through 20 (worst). The color indicates the tool that performed the gene prioritization. At rank 20, all tools arrive at their total recall of 308 causal genes. The total number of detectable causal genes is 308 because three of the 305 patient cases were affected by two disease causing genes

The results from the clinical exome interpretation simulation are shown in Figure 3. We plotted the cumulative number of detected causal genes starting from rank 1 (best but least detected) through rank 20 (worst but all detected). At rank 20, all tools arrive at their total recall of 308 causal genes. Initially, AMELIE performs the best with 166 detected causal genes at rank one, though most tools quickly catch up when considering further ranks.

**FIGURE 4:**
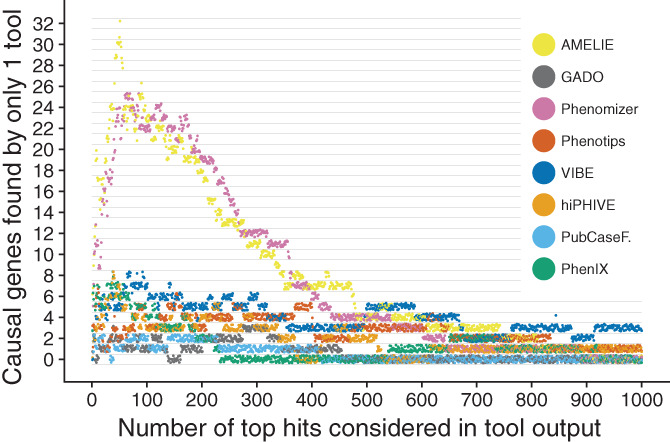
Comparison of unique hits for all tools at various cutoffs. Each dot represents the relation between number of top hits considered and how many causal genes were uniquely detected by one tool in those hits, for all patient cases. For instance, when considering the top 20 results returned by each tool, the output of VIBE includes six causal genes that were not returned by any other tool in their top 20. The color indicates the tool that performed the gene prioritization. The dots have been jittered to minimize overlap

We also investigated to which degree the assessed tools are complementary. This was achieved by counting how often a causal gene was listed within the top 20 results for each case by one or multiple tools. Remarkably, each tool listed at least one causal gene that was not listed by any of the other tested tools using this cutoff. GADO uniquely prioritized 1 causal gene, PubCaseFinder 1, Phenotips 2, hiPHIVE 3, PhenIX 5, VIBE 6, Phenomizer 15, and AMELIE 17. In total, 50 genes were listed uniquely by one tool. If we consider nonuniquely listed genes, that is, listed by one or multiple tools in their top 20, we find 121 genes. See Figure 4 for a comparison of unique hits for all tools at various cutoffs.

## 4 DISCUSSION

VIBE seems to be a solid choice for routine genome diagnostics as a phenotype‐based gene prioritizer, especially when it needs to be integrated in an automated bioinformatics pipeline. Locally installed software such as VIBE ensures availability and reproducibility. It can therefore be integrated into critical business processes, while external services such as AMELIE may offer valuable information but cannot always be used since privacy and availability are not guaranteed.

In our simulation, the tools with a high recall (AMELIE, Phenotips, PhenIX, hiPHIVE, GADO, and VIBE) were all able to prioritize the majority of causal genes within the top 5 (see Figure 3). VIBE's performance appears especially consistent as shown by the stable number of unique hits throughout a large threshold range (see Figure 4) and narrower distribution of causal gene ranks than those of comparable tools (see Figure 1, *Y*‐axis). However, VIBE does seem to be lacking in exceptionally well prioritized genes near the bottom of the graph. Looking closer into the results, we find certain genes over‐represented at high positions. For instance, in the top 10 genes of the 305 cases, we find 226 occurrences of NCBI gene 4204 (MECP2) and 219 occurrences of NCBI gene 8085 (KMT2D). This is likely caused by a form of bias, which we naturally aim to resolve in upcoming versions to let VIBE reach its fullest potential.

The question of which is the best tool is difficult to answer because of large diversity in scope, design, output size, recall rate and ultimately, user requirements. Indeed, we observed clear differences in number of genes returned, number of missed genes, and ranking dissimilarity (see Figures 1 and 2). The benefit of this diversity is complementarity. When taking the top 20 all tools together, they list 121 of 308 (39.29%) of causal genes, and of those 121, 50 were in fact unique to one tool (41.32%). In fact, unique detection occurs at nearly any cutoff, for any tool, as shown in Figure 4. The tested tools tend to each contribute unique pieces to the diagnostic puzzle. Therefore, we envision future projects that will try to combine the best features of these tools.

For now, a combination of tools could maximize chances of success. For instance, in a genome diagnostic setting with unsolved rare disease patients, it would be most efficient to first investigate candidate DNA variants in the output of a tool that returns few usual suspect genes, before progressively broadening the search to include more unexpected genes. By employing the strengths of each tool appropriately, time can be saved on easily resolved cases allowing more time to also reach a diagnosis for difficult, time‐consuming cases. Furthermore, our benchmark may be representative of clinical practice to a degree but does not demonstrate individual strengths of the tested tools. For instance, GADO is trained on gene expression data and its true strength is being able to implicate completely novel coding and noncoding genes to human phenotypes for unsolved difficult cases. In that light, it is noteworthy that GADO's performance in this solved‐case benchmark is still quite close to tools based on more direct evidence such as literature.

Finally, it must be emphasized that tools with high output volumes are still useful. Of course no clinician will examine thousands of prioritized genes based on a phenotype match. In practice, only those genes in which variants have been found and have a possible molecular effect (eg, low population frequency and high conservation) need to be followed up. Therefore, it is the variant selection step that mainly determines how many genes require further investigation, not the gene prioritization tools. A large volume of prioritized genes is translated to a small set of genes for which candidate variants were detected. As long as gene prioritizers generally rank causal genes higher than noncausal ones, they will aid the diagnostic process. We have demonstrated this point in our clinical exome interpretation simulation and visualized the results in Figure 3.

## 5 CONCLUSION

We have developed VIBE, a phenotype‐based gene prioritization tool that is straightforward to install and run locally on the command‐line, exhibits consistent performance, and therefore is a free and open‐source software (FOSS). This makes VIBE ideal for use in bioinformatic pipelines in the settings that mandate high availability and reliability. VIBE will be updated to work with upcoming DisGeNET‐RDF data releases to continue offering the latest gene‐disease associations. Currently, VIBE version 2.0 has been released, with next versions under active development. Project source code, documentation, benchmark and executables are available at https://github.com/molgenis/vibe.

## CONFLICT OF INTEREST

The authors declare that they have no competing interests.

## AUTHOR CONTRIBUTIONS

**Sander van den Hoek:** Conceptualization; writing‐review and editing. **Freerk van Dijk:** Conceptualization; writing‐review and editing. **Dennis Hendriksen:** Writing‐review and editing. **Cleo van Diemen:** Writing‐review and editing. **Lennart Johansson:** Writing‐review and editing. **Kristin Abbott:** Writing‐review and editing. **Patrick Deelen:** Writing‐review and editing. **Birgit Sikkema‐Raddatz:** Writing‐review and editing.

### PEER REVIEW

The peer review history for this article is available at https://publons.com/publon/10.1002/ggn2.10023.

## Supporting information


**Data S1** Processing of patient benchmark data. Document providing details on the data processing of the patient cases reported by Trujillano et al used in the benchmark.

Transparent‐Peer‐Review‐Record

## Data Availability

VIBE executables, documentation, source code and data resources are available at https://github.com/molgenis/vibe. Presented here is VIBE v2.0, Git commit: 934b26a5c8d12fbe36e8ef63da945eae21217bfb. The benchmark and other supporting code is available at https://github.com/molgenis/vibe-suppl and https://github.com/svandenhoek/query phenomizer. Benchmark data has been published online.^56^
