# Supplementary material for: A pipeline‐friendly software tool for genome diagnostics to prioritize genes by matching patient symptoms to literature
Source: Adv Genet (Hoboken). 2020 Aug 10;1(1):e10023. doi: 10.1002/ggn2.10023 (PMC9744518; doi:10.1002/ggn2.10023)
Supplement: Supplementary file 1 — Data S1 Processing of patient benchmark data. Document providing details on the data processing of the patient cases reported by Trujillano et al used in the benchmark. [file GGN2-1-e10023-s002.pdf]

Supplementary File 1, part of “A pipeline-friendly software tool for genome diagnostics to prioritize genes by matching patient symptoms to literature” (van der Velde *et al*. 2020)

The benchmark data consists of phenotypes and causal genes reported by Trujillano *et al*. (doi: 10.1038/ejhg.2016.146) and was retrieved from: https://www.ncbi.nlm.nih.gov/pmc/articles/PMC5255946/bin/ejhg2016146x3.docx. Alternative download location at publisher’s website: https://static-content.springer.com/esm/art%3A10.1038%2Fejhg.2016.146/MediaObjects/41431_2017_BFejhg2016146_MOESM18_ESM.docx.

Because the dataset could not easily be used for application benchmarking in its retrieved form, several adjustments were made. First, it was converted into a TSV file by copypasting the whole table from LibreOffice Writer to Calc. After this, adjustments to the data itself were made. All columns except for the LOVD, gene, OMIM info, significance and HPO terms were removed. For LOVDs with multiple genes, a separate line per gene was created with all the other information being duplicated. If an LOVD had separate information for the mother and father, the highest significance was kept (as columns with unique information regarding the father and mother were already excluded). Note that this information was not used in the tool benchmark itself. After this, multiple regular expressions were used within Atom to replace a certain string of characters with another. The regular expression " ?\t ?" was used with the replacement regular expression "\t" and then the regular expression "(\tL?P\t.+), ?" was used multiple times (until no more replacements could be made) with the replacement regular expression "$1;". As these replacements also replaced characters within phenotype names, affected terms were restored back to their original name (see Table 1). HPO terms within the data that were obsolete or that did not occur within the HPO database (for example due to using abbreviations) were replaced with their correct terms (see Table 2). For consistency, LOVD 00080824 and 00081069 were removed so that all used LOVDs had an OMIM code (as these two used a PMID code instead). Finally, the header was renamed from "LOVD patient ID\tGene\tOMIM description_new/Pubmed Description (OMIM/Pubmed Id)\tSignificance\tHPO terms" to "lovd\tgene\tomim\tsignificance\thpo_terms".

Table 1

**HPO term reported by Trujillano *et al.* as affected by regular expression**	**Restored HPO term**
Intellectual disability;severe	Intellectual disability, severe
Spontaneous;recurrent epistaxis	Spontaneous, recurrent epistaxis
Primitive reflexes (palmomental;snout;glabellar)	Primitive reflexes (palmomental, snout, glabellar)
High;narrow palate	High, narrow palate

Table 2

**HPO term reported by Trujillano et al.**	**HPO term chosen as benchmark input**	**Explanation**
Enlarged kidneys	Enlarged kidney	singular form is used in HPO
Platyspondyly (childhood)	Platyspondyly	old name is obsolete and replaced with new name
Abnormality of the lung	Abnormality of the lungs	plural form is stored as synonym
Prominent epicanthal folds	Epicanthus	old name is obsolete and replaced with new name
febrile seizures	Febrile seizures	first character to uppercase (1 of 3 occurences did not have this)
Decreased activity of m complex IV	Decreased activity of mitochondrial complex IV	used official name without abbreviation of "mitochondrial" to "m"
Abnormality of cardiac atrium	Abnormality of cardiac atrium morphology	very similar to known synonym
Abnormality of the globus pallidus	Abnormal globus pallidus morphology	very similar to known HPO name
Dilatation of the ascending aorta	Ascending tubular aorta aneurysm	old name is obsolete and replaced with new name
Primitive reflexes (palmomental, snout, glabellar)	Palmomental reflex;Snout reflex;Glabellar reflex	split up into individual names
m respiratory chain defects	Mitochondrial respiratory chain defects	used official name without abbreviation of "mitochondrial" to "m"
Abnormality of m metabolism	Abnormality of mitochondrial metabolism	used official name without abbreviation of "mitochondrial" to "m"
m myopathy	Mitochondrial myopathy	used official name without abbreviation of "mitochondrial" to "m"
Abnormality of the coronary arteries	Abnormal coronary artery morphology	very similar to known HPO name
