# Supplementary material for: A pipeline‐friendly software tool for genome diagnostics to prioritize genes by matching patient symptoms to literature
Source: Adv Genet (Hoboken). 2020 Aug 10;1(1):e10023. doi: 10.1002/ggn2.10023 (PMC9744518; doi:10.1002/ggn2.10023)
Supplement: Supplementary file 2 — Transparent‐Peer‐Review‐Record [file GGN2-1-e10023-s001.pdf]

Advanced Genetics

A pipeline-friendly software tool for genome diagnostics to prioritize genes by matching patient symptoms to literature

K. Joeri van der Velde, Sander van den Hoek, Freerk van Dijk, Dennis Hendriksen, Cleo C. van Diemen, Lennart F. Johansson, Kristin M. Abbott, Patrick Deelen, Birgit Sikkema-Raddatz and Morris A. Swertz*

*Corresponding author

Review timeline	Submission date: 10/14/2019<br>1st Editorial Decision: Major Revision – 01/07/2020<br>1st Revision received: 01/23/2020<br>Accepted: 03/20/2020

Editor: Alison Liu

1st Editorial decision	01/07/2020

Editorial Recommendation:

The authors reported a software that is open-resource and can be installed locally for helping the clinical diagnosis of genetic disease through ranking the candidate genes associated with diseases symptoms. It is a useful and convenient addition to the current online tools.

However, a revision is needed to clarify the issues raised by the reviewers. These reviews are engaging and constructive, so it is important for authors to address all reviewer comments in a point-by-point response. Particularly, please describe the algorithm thoroughly and explain how the causative genes are prioritized or ranked and the advantage over DisGeNET. As reasoned by all the reviewers, it makes more sense to use absolute ranking rather than relative ranking (you may include both, however) when you compare VIBE with other methods, i.e. considering to validate your method by focusing on the top 10 genes (practically, most meaningful for clinicians). You might want to include the Exomizer resource in the ranking process. It is also important to indicate how the HPOs are related to the diseases associated with candidate genes. For the long-term maintenance of the software, please add a plan for updating the resource. Please also update your reference, including the most recent publications in this area.

We apology the delay of our decision due to the holiday. If you have any question, please feel free to contact me. We value feedback from authors and referees alike. Thank you very much for publishing your research with the GGN.

Alison Liu, Editor
Genetics & Genomics Next

1st review	01/07/2020

Reviewer(s)' Comments to Author:

Reviewer: 1
The authors present well designed, easy to install, open-source software that addresses many of the needs of the clinical genetics community and bioinformaticians building pipelines to support them.

Major comments
The relative rank explanation in Fig 1 makes no sense to me. It assumes that each of the 10 WES filtered variants has a gene result in tools 1 and 2 i.e. the top 4 FP variants shown on the right hand side for tool 1 could have no gene-phenotype result and then the causal variant would have been ranked top, massively outperforming tool 2. For the tools such as Phenomizer that return a sensible number of candidate genes of ~100 this effect is much more likely to happen than for VIBE where ~10,000 candidate genes are returned. If Phenomizer returned the causal gene with an absolute rank of 50 out of 100 and VIBE returned it as 5000 out of 10,000 and you then ranked 10 filtered variants from a WES it is much more likely that the Phenomiser-based method ranks the correct variant as the top hit with the other 9 variants having no phenotype score or a lower score.

This same issue affects all the other analysis e.g. Figure 3. This is comparing a list of ~100 genes from Phenomiser with ~10,000 from VIBE and asking which one does a better job of finding the TP in the top 25% which is not a fair comparison as it is clearly a lot easier to find the correct answer in a list of 2500 genes rather than 25. To make if fair a score should be generated for all ~20k human genes e.g. assign a score of 0 for genes that are not returned. This is precisely why GADO achieved the best performance in this benchmarking method as it returns 100,000 genes on average.

These issues make it impossible for me to really assess if the software performs and is going to be useful for my research. This is a shame as this is a nicely presented piece of software.

Minor comments

There is an excellent introduction to the whole field and some of the many limitations of the numerous gene-based or variant-based prioritisation tools that have been published over the last few years. I concur that the majority of these tools are impossible to get hold of as they are either not open-source, don’t work, are no longer available or not updated. On the latter, how will the authors address long term maintenance?

The authors had to manually update some legacy HPO annotation to the latest version. It would be good in future versions of the software to handle this automatically using the deprecated history that is generally available in the HPO ontology file.

Reviewer: 2
Recommendation: major changes required

This manuscript describes a method for prioritizing disease genes
using the DisGenNet gene-disease association database. In contrast to
methods such as Exomiser and Phenomizer is uses text mined
gene-disease associations (similar to Amelie). The method is potentially a useful addition
to the set of tools used, but unfortunately the algorithm is not
described, and the evaluation may be set up to penalize other tools.

If these issues are addressed then this paper and its accompanying software may be a useful and
impactful part of the gene/variant prioritization landscape.

MAJOR ISSUES:

1. The VIBE algorithm is under-described.

There are in the weeds details on command line settings that are not necessary to describe in the main
paper. Instead the focus should be on describing the algorithm at a high level.

It's not clear how the input HPO IDs are used in the GDA_Max algorithm.

Also it's not even clear which of the algorithms (e.g. GDA_Max?) was used in the
evaluation.

2. The evaluation is flawed as it is not comparing absolute ranks.

You should compare the absolute ranking of the disease gene in the
sorted list of candidates. You are comparing fractions of the total
list of genes exported which will favor tools such as GADO that
provides a ranking for all genes vs more conservative tools such as
phenomizer. This is also just not a realistic clinical scenario, where
absolute ranking is most important.

E.g on figure 3, at the 25% cutoff, we can tell that GADO has the
correct gene in the top 5000 or so (assuming 20k genes returned) 85%
of the time, whereas Phenomizer has the correct gene in the top 25 or
so (assuming 100 genes returned, by your figures) >20% of the
time. This is not a meaningful comparison, and Phenomizer may well be
doing better on getting the correct gene in the top 100 or top 10 or
even top hit.

Note also that there is no need for tools to arbitrarily make higher
precision rankings (as stated in the manuscript), as there are methods to deal with genes ranked
identically (e.g. averaging or maxing).

3. DisGenNet scores may be boosted by the publication of the original benchmarks

There is also the possibility of circularity. The original EJHG paper was out in 2016, and because DisGenNet is built by mining the literature, the original benchmarks may have been ingested, meaning it's a simple lookup exercise. The correct test is to use either DisGenNet prior to this paper, or to filter out in some other way.

This is mentioned in the discussion but no steps were taken to correct or investigate if this were the case. This should be possible by sampling the correctly solved cases and investigating which papers contributed.

4. Benchmark file not available.

The file benchmark_data.tsv does not exist either in github or as a supplementary material. Without this it's impossible to replicate the analysis.

Minor related note: For github I recommend linking to a specific version, and possible syncing with zenodo.

5. Exomiser not included in comparison

The Exomiser uses similar algorithms to Phenomiser, but also makes use of model organism genes and protein interactions. The exomiser also uses genomic information but this could be omitted to compare only phenotype-based comparison.

Reviewer: 3

The manuscript describes a new tool “VIBE” to prioritise genes upon phenotypic information (HPO terms) based on DisGeNET gene-disease associations. The manuscript reads well and although the concept is not novel (similar tools are available), VIBE uses a different approach that can be easily integrated into routine genomic analysis workflows. The tool is free and open source and can be easily installed and run locally.

I have several comments and suggestions:

1) General comments:

a. It is not clear how genes have been prioritised- default parameters with GDA_max?

b. from the VIBE output and overall results it is also not clear in which score ranges (GDA, DSI) the causative genes fall.

c. A recommendation on score thresholds would also be very useful, for example a GDA above XX generally underlines a good candidate (e.g XX% of causative genes above GDA XX).

d. Not clear if any of the scores give information on the symptoms-disease-gene association. Meaning that current scores seem to inform about gene-disease associations but not how well the patient symptoms (HPOs) match the disorder associated to a specific gene.
e. Number of genes to consider are given in percentage (%- relative), for a fairer comparison, results should be taken in a top 5 / 10 rank as average number of returned genes is very different from 100 (PhenoTips) to 100000 (GADO). Comparison are then taken with 2500 (25% of average output of 10000) genes for VIBE vs 25 for Phenomyzer! Also at the practical and interpretation level, geneticists/clinical researchers might not investigate genes ranked outside the top 5/ 10 results. Or at least show results from both approaches absolute and relative.
2) Include information about disorders / cluster results: Algorithms perform differently by type of disorders. This might be something worth investigating in your cohort or to state which types of diseases (in a broad sense, e.g neurological disorders, etc.) have been included for this analysis.
3) Figure 1. It is not clear if tool 1 and tool 2 are the same or not: VIBE? Also not very clear how the information is integrated, how the gene list generated by the tools and based on HPO terms is integrated into the filtering step? Is it something manual? Is there any way to include the information at the WES filtered variant level (vcf?). It is not clear how this Figure explains that all results should be shown in a relative ranking instead of an absolute ranking.
4) Figure 2: not clear what does the relative rank represents- best or worst in which sense? I understand it might be the position of the causative gene among the top 25% considered? Or the whole output?
5) Figure 3 and 4: same as point 1- would be better to assess absolute comparison based on a specific number of genes instead of an output fraction taking into account that output sizes are very different. At least show and compare both approaches: absolute and relative.
6) Table 1: ADCK3 should be removed from this table as it is a nomenclature issue that should be resolved before running these tools. Is there any explanation why VIBE could have missed those cases? Atypical phenotype?
7) Suggestion: include as input ORDO terms. This is something DisGeNET should enable and that might be very useful for well-defined types of disorder.
8) Discussion:
a. “we would encourage to using the latest approved gene symbols”. This would depend on the assembly used for genomic analysis, therefore what should be encouraged is to check that the HGNC version used to annotate genomic data is the same as the one downloaded in the TBD; otherwise symbols should be checked and changed (e.g Biomart) accordingly in order to avoid any misdiagnosis. Idem with HPO terms, input and TBD version should be compared before performing any analysis.
b. Related previous comments: “we assessed gene prioritization performance by relative ranking instead of absolute ranking for reasons explained in Figure 1.” This is really not clear. I think that both approaches should be shown.

Reviewer: 4
The authors present VIBE, a phenotype prioritization program that performs computational differential diagnosis by taking a list of HPO terms and returning a file with prioritized genes and diseases.

Major comments

1) The authors provide a short description of the input and output formats but they do not provide much description of the actual method by which VIBE ranks diseases. The tool appears to be using metrics from DisGeNET. However it is unclear to me how if it is different from DisGeNET. Why should users choose VIBE as opposed to using the DisGeNET API? Phenotype data alone is not identifying (outside of perhaps edge cases).

2) The authors present their results as the top 25% output fractions. This does not seem to be fair, because the different tools return vastly different total numbers of genes in their hands "For Phenomizer, the average number of returned genes was just over 10e2 , for PhenoTips this was just over 10e3 , for AMELIE and VIBE around 10e4 , while GADO was close to 10e5"
It would be better to report the number of times a tool placed the correct diagnosed ion the first k ranks for k=1,2,..., 10. It is unlikely that a clinical will look much further down than rank 10, and so being in the top 25% of 10,000 genes is not a good way to validate a tool!
Also, what is the reason for the discrepancy in the number of genes being returned?

3) However, despite explosive data growth[3] and time- consuming best efforts, chances of succesfully detecting a causal variant are 30% at best[4–6].
=> This is not true. There are some studies with higher pickup rates. One recent review article gives a figure of around 40% (Wright CF, FitzPatrick DR, Firth HV. Paediatric genomics: diagnosing rare disease in children. Nat Rev Genet. 2018 May;19(5):253-268.)
=> I found this a little confusing because after this introduction I was expecting a tool that would analyze VCF files plus phenotypes. The figure of 30% does not apply to clinical only differential diagnosis. Can the authors clarifiy?

Minor
1) The authors should describe their plans for keeping the resource up to date. How often will new data files be made available?
2) Will the code run on Java 11 or more modern Java versions?

Reviewer: 5
VIBE: a pipeline-friendly software tool for genome diagnostics to prioritize genes by matching patient symptoms to literature

This manuscript reports on the development of a new phenotype-based variant prioritization software application for deployment within clinical systems. While a number of such tools exist, the authors argue that none are both deployable locally in clinical variant prioritization pipelines and have ready access to the current biomedical literature. While VIBE may offer some advances over current technologies by using DisGeneNet and/or its specific algorithms, it is certainly not the case that most tools cannot be deployed locally – most clinical interpretation pipelines pull together a variety of such tools, and secondly, the lack of robust evaluation measures diminish confidence in the potential advances.

First, the background does not describe the state of the art in the use of computable phenotype data in exome analysis and the diversity of algorithms nor the challenges in use of text mined content in such circumstances. Since the main advance of the paper is the use of text-extracted phenotypes within DisGeneNet in the context of exome analysis, it would, it would seem necessary to first explain how these phenotype algorithms function (largely ontology-based algorithms for inexact phenotype matching).

Second, the article also does not cite the most recent or some of the most relevant literature. There is not a single citation from 2019, a year with numerous tools and their applications in the area of phenotype-driven diagnostics, as well as a variety of graph based computational methods for patient stratification and clustering for diagnostic or other precision-medicine purposes.

Third, in terms of evaluation, there are a number of manuscripts detailing different methods for evaluating variant prioritization that include spiking exomes with correct or incorrect variants, adding phenotype noise, removing candidate diagnoses, removing specific data sources or pathogenicity measures, etc. It does not seem as though any of these types of approaches for evaluating the robustness of the candidate results in the face of real-world variability have been applied in the evaluation of VIBE. In fact, I could not really determine without a lot of investigation in the associated github (thank you for your open science, though!) whether the patient exomes were used or simply candidate genes or ?? the actual pipeline is not adequately described in the manuscript, in any case.

Fourth, in looking at the patients used in the benchmarking, it seems as though text matching was used to encode HPO terms, but no identifiers are provided and the version of the HPO used in the benchmarking is pointed at the always-latest release. This can lead to spurious/different results This methodology documentation could be made more robust, and a specific version of the HPO used in the analysis should be indicated.

Fifth, the choice of tools to benchmark against is a bit outdated. Phenomiser was developed by the HPO team years ago and has largely been replaced with Exomiser, which is the deployed tool within numerous clinical diagnostic pipelines such as in Genomics England, Undiagnosed Disease Network, etc. The HPO website (https://hpo.jax.org/app/tools/external) and recent manuscript (see https://doi.org/10.1093/nar/gky1105) both provide a list of external tools and citations that would be worth investigating. It is true that Amelie – probably the most similar tool - does not function as a locally deployable stack (so far as this reviewer knows), this is a valid criticism. Another relevant tool is PubCaseFinder. Another example is Saklatvala et al https://www.ncbi.nlm.nih.gov/pubmed/29460986. A more thorough review of tools and literature and their specific functionality would help identify the most appropriate resources to benchmarking against and the best methods for doing so.

Sixth, another important aspect that is omitted in mining the literature that is not discussed is that the biomedical literature does not generally contain the full set of phenotypes for a specific disease or patient or cohort. This information is often curated from textbooks or directly from clinical geneticists.

Other sources of genotype-phenotype information are through direct submission to databases such as Clinvar, though the robustness is fairly minimal for ClinVar. Efforts in the GA4GH and ClinGen really aim to improve direct knowledge capture and sharing to complement what is underpopulated in the literature. While DisGeneNet may aim to address the combinatorial issues, relying on one integrative source without an understanding of these issues or the ability to configure against them could lead to spurious or omitted results.

Seventh, the candidate diagnoses come seemingly from OMIM and MeSH. It is well known that there are many Mendelian diseases not in MeSH, that the MeSH disease hierarchy is not an adequate computational representation, and there are a number of additional sources of disease information that may provide different results. It is not clear if additional disease-gene sources are included within DisGeneNet, are used for the prioritization, but then not reported in the candidate results? Why would DisGeneNet use MeSH for example to reveal disease pleiotropy? I would be very careful with such a measure and base it on a more robust disease terminology and its associations (or perhaps I misunderstood).

Eighth, this reviewer concurs regarding the use of current gene nomenclature and with the frustrations that come from this issue. However, any good pipeline should be able to track provenance of gene nomenclature in data sources, flag issues, and convert to current nomenclature and identifiers using a variety of APIs and services, for example MyGene.info.

In summary, VIBE poses promising use of the integrated literature and knowledge within DisGeneNet in the context of variant prioritization, but perhaps the focus should be on its incorporation into existing pipelines and tools as a corroboration feature rather than a standalone too. Most clinical pipelines leverage a multiplicity of algorithms and methods and the best methods for integration of DisGeneNet might be the win here.

1st Author Response to Reviewers and Editor	01/28/2020

Response: We thank all reviewers for their kind words and constructive criticisms. We have addressed all points and believe that the quality of the paper has now significantly improved. We would like to begin our response by addressing a key issue that was raised by all reviewers.

Key issue: The relative versus absolute ranking in the benchmark.

While we still think there is some validity to assess ranking performance in a relative way, for instance in unsolved case exome analysis where a huge number of genes is prioritised independently from DNA variants, we do agree with all reviewers that this was not the best nor the most intuitive way to present the results. This way of scoring also could indeed be inflated with zeroes to artificially boost tools with less output, and while we of course tried to avoid such bias, this issue is still a valid concern. In addition, we agree that for many clinical and research use cases, the top-N output genes will be investigated, in which case tools with fewer output and better absolute ranking are definitely preferred.

Taking all of this into consideration, we have chosen to now present the primary benchmark results in

absolute terms and present the output of the tools ‘as is’. Figure 1 shows that each tool has its own output size and ranking distribution. Figure 2 shows a heatmap and clustering of absolute ranking for each tool to make visually clear that the tools each have their own way of ranking.

To provide a more realistic picture of how the tools would behave in a practical setting, we have now added a simulation of clinical exome interpretation. Here we demonstrate what happens when the tools were asked to rank causal genes within sets of 19 other random clinical genes. The results are shown in Figure 3. We added details and explanations on this analysis in the “Patient benchmark” (p.3), “Results” (p.3) and “Discussion” (p.4) sections.

Lastly, we checked the number of uniquely solved cases by one tool against absolute cutoffs and reported the findings. This demonstrates that all tools are complementarity to each other, because each tool typically provides a unique piece of the diagnostic puzzle. To emphasize this point, we plotted the relation between cutoff and uniquely detected causal genes in Figure 4 and further highlight tool complementarity in the “Results” (p.3) and “Discussion” (p.4) sections. From this we conclude that we should perhaps work towards a tool that combines the strengths of each tool.

Reviewer: 1

Comment: The authors present well designed, easy to install, open-source software that addresses many of the needs of the clinical genetics community and bioinformaticians building pipelines to support them.

MAJOR ISSUES

Comment: The relative rank explanation in Fig 1 makes no sense to me. It assumes that each of the 10 WES filtered variants has a gene result in tools 1 and 2 i.e. the top 4 FP variants shown on the right hand side for tool 1 could have no gene-phenotype result and then the causal variant would have been ranked top, massively outperforming tool 2. For the tools such as Phenomizer that return a sensible number of candidate genes of ~100 this effect is much more likely to happen than for VIBE where ~10,000 candidate genes are returned. If Phenomizer returned the causal gene with an absolute rank of 50 out of 100 and VIBE returned it as 5000 out of 10,000 and you then ranked 10 filtered variants from a WES it is much more likely that the Phenomiser-based method ranks the correct variant as the top hit with the other 9 variants having no phenotype score or a lower score.

This same issue affects all the other analysis e.g. Figure 3. This is comparing a list of ~100 genes from Phenomiser with ~10,000 from VIBE and asking which one does a better job of finding the TP in the top 25% which is not a fair comparison as it is clearly a lot easier to find the correct answer in a list of 2500 genes rather than 25. To make if fair a score should be generated for all ~20k human genes e.g. assign a score of 0 for genes that are not returned. This is precisely why GADO achieved the best performance in this benchmarking method as it returns 100,000 genes on average.

These issues make it impossible for me to really assess if the software performs and is going to be useful for my research. This is a shame as this is a nicely presented piece of software.

Response: We agree with these issues and have addressed them. For our detailed response on this, please see "Key issue: The relative versus absolute ranking in the benchmark" in the opening statement addressed to all reviewers.

MIMOR COMMENTS

Comment: There is an excellent introduction to the whole field and some of the many limitations of the numerous gene-based or variant-based prioritisation tools that have been published over the last few years. I concur that the majority of these tools are impossible to get hold of as they are either not open-source, don't work, are no longer available or not updated. On the latter, how will the authors address long term maintenance?

Response: Long term maintenance is indeed an issue in this field. In the case of VIBE, there are two aspects to this. First is the DisGeNET source data. DisGeNET seems to be a stable and well cited resource. Currently it is at release 6.0, and of course we hope to see more releases in the future so that VIBE may benefit, but of course this is ultimately out of our control. However, the VIBE database has its own download that does not depend on DisGeNET availability and even if DisGeNET would not longer update its resource, the VIBE tool can still be improved, and/or updated to use alternative resources, or perhaps even use custom-built resources that are created by us or others using the DisGeNET approach.

The second are updates of the VIBE tool itself. The code and documentation are and will remain open source under the GNU Lesser General Public License v3.0 license, so in principle, maintenance could become a community effort. In case that does not happen, VIBE is currenly at version 2.0 which was already a significant improvement over 1.0, and version 3.0 is currently being developed by our team. Given the importance of such tools, in general but also for our clinic, we surely expect to continue work. We have updated the "Conclusion" (p.5) section with our clear intention to maintain VIBE and keep it up to date. Next practical steps include adoption in our own local bioinformatic pipelines and diagnostic practice in combination with other tools. We would like to note that even without updates, VIBE will run on systems for many years to come because it is released as a standalone executable that does not depend on any external libraries, so it will run on any operating system as long as Java 8+ is available, avoiding dreaded "dependency hell".

Comment: The authors had to manually update some legacy HPO annotation to the latest version. It would be good in future versions of the software to handle this automatically using the deprecated history that is generally available in the HPO ontology file.

Response: We agree that this could be an issue. Therefore we created a Github issue that will be addressed in future versions of VIBE. Please see: https://github.com/molgenis/vibe/issues/25.

Reviewer: 2

Comment: Recommendation: major changes required

This manuscript describes a method for prioritizing disease genes using the DisGenNet gene-disease association database. In contrast to methods such as Exomiser and Phenomizer is uses text mined gene-disease associations (similar to Amelie). The method is potentially a useful addition to the set of tools used, but unfortunately the algorithm is not described, and the evaluation may be set up to penalize other tools.

If these issues are addressed then this paper and its accompanying software may be a useful and impactful part of the gene/variant prioritization landscape.

MAJOR ISSUES:

Comment: 1. The VIBE algorithm is under-described.

There are in the weeds details on command line settings that are not necessary to describe in the main paper. Instead the focus should be on describing the algorithm at a high level.

It's not clear how the input HPO IDs are used in the GDA_Max algorithm.

Response: We fully agree with this issue and have added a new “Algorithm” (p.2) section in the text that should explain the workings of VIBE in detail. In addition, we have also expanded the “Implementation” (p.2) section with detailed information on how the database was created and how it can be re-created.

Comment: Also it's not even clear which of the algorithms (e.g. GDA_Max?) was used in the Evaluation.

Response: This was indeed unclear. We have clarified which tools and settings were used in the “Patient benchmark” (p.3) section. In the case of VIBE, there is no longer a question which algorithm was used since in version 2.0, only GDA_Max is available, because it simply works better than DSI or DPI under any circumstance. To make things faster and easier, we removed DSI and DPI from the tool implementation and cmdline options in version 2.0 (the version presented in the manuscript).

Comment: 2. The evaluation is flawed as it is not comparing absolute ranks.

You should compare the absolute ranking of the disease gene in the sorted list of candidates. You are comparing fractions of the total list of genes exported which will favor tools such as GADO that provides a ranking for all genes vs more conservative tools such as phenomizer. This is also just not a realistic clinical scenario, whereabsolute ranking is most important.

E.g on figure 3, at the 25% cutoff, we can tell that GADO has the correct gene in the top 5000 or so (assuming 20k genes returned) 85% of the time, whereas Phenomizer has the correct gene in the top 25 or so (assuming 100 genes returned, by your figures) >20% of the time. This is not a meaningful comparison, and Phenomizer may well be doing better on getting the correct gene in the top 100 or top 10 or even top hit.

Response: We agree with these issues and have addressed them. For our detailed response on this, please see "Key issue: The relative versus absolute ranking in the benchmark" in the opening statement addressed to all reviewers.

Comment: Note also that there is no need for tools to arbitrarily make higher precision rankings (as stated in the manuscript), as there are methods to deal with genes ranked identically (e.g. averaging or maxing).

Response: Good point. We agree and have removed this moot statement from the “Discussion” (p.4) section.

Comment: 3. DisGenNet scores may be boosted by the publication of the original benchmarks

There is also the possibility of circularity. The original EJHG paper was out in 2016, and because DisGenNet is built by mining the literature, the original benchmarks may have been ingested, meaning it's a simple lookup exercise. The correct test is to use either DisGenNet prior to this paper, or to filter

out in some other way.

This is mentioned in the discussion but no steps were taken to correct or investigate if this were the case. This should be possible by sampling the correctly solved cases and investigating which papers contributed.

Response: We also believe that any circular reasoning must be avoided for a fair benchmark. Therefore, a check was done on the “pubmed.ttl” file of the DisGeNET RDF data to check for the existence of the pubmed ID from which the benchmark data was generated. This pubmed ID was not present in the pubmed.ttl of either DisGeNET r5 or r6. To be precise: grep "27848944" pubmed.ttl (for disgenetv5.0-rdf-v5.0.0 and disgenetv6.0-rdf-v6.0.0) yielded 0 matches. We have removed this issue from the “Discussion” (p.4) section and have added this check to the “Patient benchmark” (p.3) section.

Comment: 4. Benchmark file not available.

The file benchmark_data.tsv does not exist either in github or as a supplementary material. Without this it's impossible to replicate the Analysis.

Response: This was indeed an oversight on our part. We have now uploaded all benchmark input and output files to Zenodo, available under http://doi.org/10.5281/zenodo.3634601. This has also been added to the manuscript under “Availability of data and material” (p.5).

MINOR ISSUES

Comment: For github I recommend linking to a specific version, and possible syncing with zenodo.

Response: We agree that linking to a specific version is good practice. In the “Availability of data and material” (p.5) section we now refer to a specific Git commit for VIBE version 2.0 (934b26a5c8d12fbe36e8ef63da945eae21217bfb).

Comment: 5. Exomiser not included in comparison

The Exomiser uses similar algorithms to Phenomiser, but also makes use of model organism genes and protein interactions. The exomiser also uses genomic information but this could be omitted to compare only phenotype-based comparison.

Response: Yes, Exomiser should have been included. To resolve this issue, we have contacted the main developer of Exomiser who was kind enough to supply us with the appriopriate Exomiser release that includes this functionality. The results of both the PhenIX and hiPHIVE prioritizers have now been included in the benchmark. Please see “Patient benchmark” (p.3) section for details on versions and settings, as well as the “Results” (p.3) and “Discussion” (p.4) section for interpretation of the overall results.

Reviewer: 3

Comment: The manuscript describes a new tool “VIBE” to prioritise genes upon phenotypic information (HPO terms) based on DisGeNET gene-disease associations. The manuscript reads well and although the concept is not novel (similar tools are available), VIBE uses a different approach that can be easily integrated into routine genomic analysis workflows. The tool is free and open source and can be easily

installed and run locally.
I have several comments and suggestions:

GENERAL COMMENTS

Comment: a. It is not clear how genes have been prioritised- default parameters with GDA_max?

Response: This was indeed unclear. We have clarified which tools and settings were used in the "Patient benchmark" (p.3) section. In the case of VIBE, there is no longer a question which algorithm was used since in version 2.0, only GDA_Max is available, because it simply works better than DSI or DPI under any circumstance. To make things faster and easier, we removed DSI and DPI from the tool implementation and cmdline options in version 2.0 (the version presented in the manuscript).

Comment: b. from the VIBE output and overall results it is also not clear in which score ranges (GDA, DSI) the causative genes fall.

Response: We now use GDA, for which possible scores range between 0 and 1. The exact score calculation can be found on https://www.disgenet.org/dbinfo#section31 and DisGeNET publications (e.g. https://academic.oup.com/nar/article/45/D1/D833/2290909) The GDA_max indicates the highest possible GDA-score found for that gene in the context of the given HPO-terms and it used to by VIBE to define its final output gene list. We have clarified how the ranking works in the new "Algorithm" (p.2) section.

Comment: c. A recommendation on score thresholds would also be very useful, for example a GDA above XX generally underlines a good candidate (e.g XX% of causative genes above GDA XX).

Response: We agree that further exploration into the scores could be beneficial. We could use the GDA scores to calculate and provide Positive Predictive Values per ranked gene instead of just ordering the hits from best to worst. For instance, based on a truth set, the Positive Predictive Value could be determined for genes in score ranges 0.0 - 0.1, 0.1 - 0.2, etc. or perhaps from a continuous value. These could be included in the VIBE output to give a sense of trustworthiness. Subsequently, the benchmark could be extended to provide a PPV for all other testable tools. In practice, a clinician may then choose which result to 'trust' in an output gene list based on this metric. We have created an issue in the VIBE Github to work on this excellent suggestion in the future, see https://github.com/molgenis/vibe/issues/33.

Comment: d. Not clear if any of the scores give information on the symptoms-disease-gene association. Meaning that current scores seem to inform about gene-disease associations but not how well the patient symptoms (HPOs) match the disorder associated to a specific gene.

Response: Yes, this was indeed a limitation in VIBE version 1.0. The GDA scores indicated how much evidence (curated, animal models & literature) there was between a UMLS disease identifier and a NCBI gene identifier. In VIBE version 2.0, we improved this process. Now, phenotypes are linked to UMLS disease identifiers (CUIs) via mapping resources that currently include DisGeNET, UMLS Metathesaurus and Orphadata's HOOM. The CUIs are then used to retrieve GDAs and their accompanying scores. Other associations to the genes are therefore now excluded. We clarified this in the new "Algorithm" (p.2) section.

Comment: e. Number of genes to consider are given in percentage (%- relative), for a fairer comparison,

results should be taken in a top 5 / 10 rank as average number of returned genes is very different from 100 (PhenoTips) to 100000 (GADO). Comparison are then taken with 2500 (25% of average output of 10000) genes for VIBE vs 25 for Phenomyzer! Also at the practical and interpretation level, geneticists/clinical researchers might not investigate genes ranked outside the top 5/ 10 results. Or at least show results from both approaches absolute and relative.

2) Include information about disorders / cluster results: Algorithms perform differently by type of disorders. This might be something worth investigating in your cohort or to state which types of diseases (in a broad sense, e.g neurological disorders, etc.) have been included for this analysis.

3) Figure 1. It is not clear if tool 1 and tool 2 are the same or not: VIBE? Also not very clear how the information is integrated, how the gene list generated by the tools and based on HPO terms is integrated into the filtering step? Is it something manual? Is there any way to include the information at the WES filtered variant level (vcf?). It is not clear how this Figure explains that all results should be shown in a relative ranking instead of an absolute ranking.

4) Figure 2: not clear what does the relative rank represents- best or worst in which sense? I understand it might be the position of the causative gene among the top 25% considered? Or the whole output?

5) Figure 3 and 4: same as point 1- would be better to assess absolute comparison based on a specific number of genes instead of an output fraction taking into account that output sizes are very different. At least show and compare both approaches: absolute and relative.

Response: We agree with these issues regarding the benchmarking and have addressed them. For our detailed response on this, please see "Key issue: The relative versus absolute ranking in the benchmark" in the opening statement addressed to all reviewers.

We also agree that it would be worth investigating whether some algorithms perform better for disease types/categories, disease subtypes, specific diseases, organ manifestation categories, perhaps even age of onset or disease severity. A quick exploration in the previous version of VIBE (v1.0) did not show any significant results but this is surely interesting to look into, therefore we have created an issue on the VIBE Github for this at https://github.com/molgenis/vibe/issues/34.

Regarding the integration into WES/VCF level, this is a good point. We have clarified in the “Background” (p.1) section that intented use of VIBE is not on VCF files directly, but rather be made part of composable analysis/interpretation pipelines. This resolves the issue of ‘monolithic’ tools try to combine many functionalities but are ultimately not able to analyse many molecular data modalities and are difficult to maintain.

Comment: 6) Table 1: ADCK3 should be removed from this table as it is a nomenclature issue that should be resolved before running these tools. Is there any explanation why VIBE could have missed those cases? Atypical phenotype?

Response: We fully agree that nomenclature issues should have be resolved. We have rectified this by converting all tool output genes to NCBI gene identifiers, and now have significantly fewer missed genes in the benchmark, showing that this indeed needed to be done. In fact, VIBE v2.0 now outputs NCBI gene identifiers to prevent such issues in the future. Since this issue no longer applies, we have removed it from the “Discussion” (p.4) section.

Comment: 7) Suggestion: include as input ORDO terms. This is something DisGeNET should enable and that might be very useful for well-defined types of disorder.

Response: We think this is a great suggestion. As of VIBE version 2.0, Orphadata's HOOM is included in the triple database to find ORDO terms that are linked to the input HPO terms. However, directly using ORDO terms as input is not yet possible. Allowing ORDO and perhaps also other types of terms as input would make a lot of sense. An issue was created with this request: https://github.com/molgenis/vibe/issues/26.

Comment:
8) Discussion:
a. "we would encourage to using the latest approved gene symbols". This would depend on the assembly used for genomic analysis, therefore what should be encouraged is to check that the HGNC version used to annotate genomic data is the same as the one downloaded in the TBD; otherwise symbols should be checked and changed (e.g Biomart) accordingly in order to avoid any misdiagnosis. Idem with HPO terms, input and TBD version should be compared before performing any analysis.

Response: We agree and as mentioned before, we have rectified this problem and made sure there will be no more issues in the future since VIBE now uses NCBI gene identifiers. These identifiers are also used internally by DisGeNET to link to CUIs (UMLS disease identifiers). Further, we have declared all used versions of data and resources in the "Implementation" (p.2) section.

Comment: b. Related previous comments: "we assessed gene prioritization performance by relative ranking instead of absolute ranking for reasons explained in Figure 1." This is really not clear. I think that both approaches should be shown.

Response: As mentioned, we agree with this issue and have addressed it. For our detailed response on this, please see "Key issue: The relative versus absolute ranking in the benchmark" in the opening statement addressed to all reviewers.

Reviewer: 4

Comment: The authors present VIBE, a phenotype prioritization program that performs computational differential diagnosis by taking a list of HPO terms and returning a file with prioritized genes and diseases.

MAJOR COMMENTS

Comment: 1) The authors provide a short description of the input and output formats but they do not provide much description of the actual method by which VIBE ranks diseases. The tool appears to be using metrics from DisGeNET. However it is unclear to me how if it is different from DisGeNET. Why should users choose VIBE as opposed to using the DisGeNET API? Phenotype data alone is not identifying (outside of perhaps edge cases).

Response: Yes, we agree that this should have been made far more explicit. Therefore, we have added the following clarification to the "Implementation" (p.2) section: "The value that VIBE adds to DisGeNET for use in genome diagnostics is that it (i) provides a quality open-source command-line executable, (ii) semantically integrates Dis\-Ge\-NET with additional resources, (iii) allows users to prioritize genes by HPO codes, and (iv) runs offline to ensure availability and reproducibility."

Comment: 2) The authors present their results as the top 25% output fractions. This does not seem to be fair, because the different tools return vastly different total numbers of genes in their hands “For Phenomizer, the average number of returned genes was just over 10e2 , for PhenoTips this was just over 10e3 , for AMELIE and VIBE around 10e4 , while GADO was close to 10e5” It would be better to report the number of times a tool placed the correct diagnosed ion the first k ranks for k=1,2,..., 10. It is unlikely that a clinical will look much further down than rank 10, and so being in the top 25% of 10,000 genes is not a good way to validate a tool! Also, what is the reason for the discrepancy in the number of genes being returned?

Response: We agree with these issues and have addressed them. For our detailed response on this, please see "Key issue: The relative versus absolute ranking in the benchmark" in the opening statement addressed to all reviewers. The differences in returned number of genes are due to the highly diverse natures of the various tested tools, which we explain in the “Patient benchmark” (p.3) section as well as the “Discussion” (p.4) section.

Comment: 3) However, despite explosive data growth[3] and time- consuming best efforts, chances of succesfully detecting a causal variant are 30% at best[4–6].
>=> This is not true. There are some studies with higher pickup rates. One recent review article gives a figure of around 40% (Wright CF, FitzPatrick DR, Firth HV. Paediatric genomics: diagnosing rare disease in children. Nat Rev Genet. 2018 May;19(5):253-268.)

Response: We thank the reviewer for this suggestion and have updated the “Background” (p.1) section accordingly.

Comment: => I found this a little confusing because after this introduction I was expecting a tool that would analyze VCF files plus phenotypes. The figure of 30% does not apply to clinical only differential diagnosis. Can the authors clarifiy?

Response: This is a good point. We have clarified in the “Background” (p.1) section that intented use of VIBE is not on VCF files directly, but rather be made part of composable analysis/interpretation pipelines. This resolves the issue of ‘monolithic’ tools try to combine many functionalities but are ultimately not able to analyse many molecular data modalities and are difficult to maintain.

MIMOR COMMENTS

Comment: 1) The authors should describe their plans for keeping the resource up to date. How often will new data files be made available?

Response: Long term maintenance is indeed an issue in this field. In the case of VIBE, there are two aspects to this. First is the DisGeNET source data. DisGeNET seems to be a stable and well cited resource. Currently it is at release 6.0, and of course we hope to see more releases in the future so that VIBE may benefit, but of course this is ultimately out of our control. However, the VIBE database has its own download that does not depend on DisGeNET availability and even if DisGeNET would not longer update its resource, the VIBE tool can still be improved, and/or updated to use alternative resources, or perhaps even use custom-built resources that are created by us or others using the DisGeNET approach.

The second are updates of the VIBE tool itself. The code and documentation are and will remain open source under the GNU Lesser General Public License v3.0 license, so in principle, maintenance could

become a community effort. In case that does not happen, VIBE is currenly at version 2.0 which was already a significant improvement over 1.0, and version 3.0 is currently being developed by our team. Given the importance of such tools, in general but also for our clinic, we surely expect to continue work. We have updated the “Conclusion” (p.5) section with our clear intention to maintain VIBE and keep it up to date. Next practical steps include adoption in our own local bioinformatic pipelines and diagnostic practice in combination with other tools. We would like to note that even without updates, VIBE will run on systems for many years to come because it is released as a standalone executable that does not depend on any external libraries, so it will run on any operating system as long as Java 8+ is available, avoiding dreaded “dependency hell”.

Comment: 2) Will the code run on Java 11 or more modern Java versions?

Response: Yes, we have tested this, and indeed it runs on Java 11. Local tests with openjdk v11 did not cause any issues. Incidentally, we are also working on Docker images for future releases, which will perhaps make it even easier to run for certain users.

Reviewer: 5

Comment: VIBE: a pipeline-friendly software tool for genome diagnostics to prioritize genes by matching patient symptoms to literature. This manuscript reports on the development of a new phenotype-based variant prioritization software application for deployment within clinical systems. While a number of such tools exist, the authors argue that none are both deployable locally in clinical variant prioritization pipelines and have ready access to the current biomedical literature. While VIBE may offer some advances over current technologies by using DisGeneNet and/or its specific algorithms, it is certainly not the case that most tools cannot be deployed locally – most clinical interpretation pipelines pull together a variety of such tools, and secondly, the lack of robust evaluation measures diminish confidence in the potential advances.

Response: There are indeed a number of alternative tools (in fact, seven of which are now included in our benchmark), some of which also deployable in a local setting. However, it is also true that there are many tools that suffer from being a remote service only, closed source, abandoned, or have simply vanished. Here we offer a tool that is open source, works offline, and is pipeline-ready, solving many of these issues, and allowing the DisGeNET resource to be used for routine genome diagnostic purposes. We have clarified these points in the “Background” (p.1) section as well as in the “Implementation” (p.2) section.

Regarding the lack of robust evaluation measures, we also agree and have addressed the benchmarking issues. For our detailed response on this, please see "Key issue: The relative versus absolute ranking in the benchmark" in the opening statement addressed to all reviewers.

Comment: First, the background does not describe the state of the art in the use of computable phenotype data in exome analysis and the diversity of algorithms nor the challenges in use of text mined content in such circumstances. Since the main advance of the paper is the use of text-extracted phenotypes within DisGeneNet in the context of exome analysis, it would, it would seem necessary to first explain how these phenotype algorithms function (largely ontology-based algorithms for inexact phenotype matching).

Response: We agree that certain aspects were under-described. We now have clarified how VIBE functions in the “Algorithm” (p.2) section and how the database was built in the “Implementation” (p.2)

section. This should provide insights how the VIBE's ontological matching works. We have updated the "Background" (p.1) section to better cover the state of the art tools. Since we are presenting a comparison of many tools that perform the same function but using very different approaches, we mention the key differences ("The scope of these tools differs..."), now clearly indicate which tool, data, versions and settings were used in the "Patient benchmark" (p.3) section, and in the "Discussion" (p.4) section we now better emphasize their complementarity.

Comment: Second, the article also does not cite the most recent or some of the most relevant literature. There is not a single citation from 2019, a year with numerous tools and their applications in the area of phenotype-driven diagnostics, as well as a variety of graph based computational methods for patient stratification and clustering for diagnostic or other precision-medicine purposes.

Review: Indeed, we did not cite the latest advancements in this field. We have now updated our citations to reference recent works where applicable. Please see the updated "Background" (p.1) section.

Comment: Third, in terms of evaluation, there are a number of manuscripts detailing different methods for evaluating variant prioritization that include spiking exomes with correct or incorrect variants, adding phenotype noise, removing candidate diagnoses, removing specific data sources or pathogenicity measures, etc. It does not seem as though any of these types of approaches for evaluating the robustness of the candidate results in the face of real-world variability have been applied in the evaluation of VIBE.

Review: Yes, we also thought that a more realistic, daily-practice-like scenario was missing. We have now added a simulation of how these gene-prioritization tools would behave in a clinical exome interpretation. We reused the patient benchmark set to (i) keep this factor consistent across the manuscript and (ii) use realistic data derived from clinical practice. This simulation is described in the "Patient benchmark" (p.3) section at "To find out how the tools would perform in a real-life scenario [...] causal genes ranked first, second, third, and so on.".

Comment: In fact, I could not really determine without a lot of investigation in the associated github (thank you for your open science, though!) whether the patient exomes were used or simply candidate genes or ?? the actual pipeline is not adequately described in the manuscript, in any case.

Response: This was indeed a bit vague. We have clarified in the "Background" (p.1) section that intented use of VIBE is not on VCF files directly, but rather be made part of composable analysis/interpretation pipelines. This resolves the issue of 'monolithic' tools try to combine many functionalities but are ultimately not able to analyse many molecular data modalities and are difficult to maintain.

Comment: Fourth, in looking at the patients used in the benchmarking, it seems as though text matching was used to encode HPO terms, but no identifiers are provided and the version of the HPO used in the benchmarking is pointed at the always-latest release. This can lead to spurious/different results This methodology documentation could be made more robust, and a specific version of the HPO used in the analysis should be indicated.

Response: We agree that versions were missing for reproducibility. We have updated the "Patient benchmark" (p.3) section to include precise versions of resources, tools, versions, and settings used to run each of the tools.

Comment: Fifth, the choice of tools to benchmark against is a bit outdated. Phenomiser was developed by the HPO team years ago and has largely been replaced with Exomiser, which is the deployed tool within numerous clinical diagnostic pipelines such as in Genomics England, Undiagnosed Disease Network, etc. The HPO website (https://hpo.jax.org/app/tools/external) and recent manuscript (see https://doi.org/10.1093/nar/gky1105) both provide a list of external tools and citations that would be worth investigating.

Response: We thank the reviewer for linking these resources. We agree that Exomiser should have been included. To resolve this issue, we have contacted the main developer of Exomiser who was kind enough to supply us with the appriopriate Exomiser release that includes this functionality. The results of both the PhenIX and hiPHIVE prioritizers have now been included in the benchmark. Please see "Patient benchmark" (p.3) section for details on versions and settings, as well as the "Results" (p.3) and "Discussion" (p.4) section for interpretation of the overall results.

Comment: It is true that Amelie – probably the most similar tool - does not function as a locally deployable stack (so far as this reviewer knows), this is a valid criticism. Another relevant tool is PubCaseFinder. Another example is Saklatvala et al https://www.ncbi.nlm.nih.gov/pubmed/29460986. A more thorough review of tools and literature and their specific functionality would help identify the most appropriate resources to benchmarking against and the best methods for doing so.

Response: We thank the reviewer for bringing these works to our attention. We have considered these tools and found that the Python script by Saklatvala et al. is no longer available under the URL mentioned in the paper (in fact, the entire subdomain https://atlas.genetics.kcl.ac.uk seems to have disappeared). We have chosen to cite this paper as an example of software "no longer being available". PubCaseFinder is certainly interesting and and we were happy to see it was available and had a web-API. Therefore, we have included PubCaseFinder in our benchmark. We now have a total of eight tools included in the benchmark which we believe is a good representation of tools in this field.

Comment: Sixth, another important aspect that is omitted in mining the literature that is not discussed is that the biomedical literature does not generally contain the full set of phenotypes for a specific disease or patient or cohort. This information is often curated from textbooks or directly from clinical geneticists. Other sources of genotype-phenotype information are through direct submission to databases such as Clinvar, though the robustness is fairly minimal for ClinVar. Efforts in the GA4GH and ClinGen really aim to improve direct knowledge capture and sharing to complement what is underpopulated in the literature. While DisGeneNet may aim to address the combinatorial issues, relying on one integrative source without an understanding of these issues or the ability to configure against them could lead to spurious or omitted results.

Response: We thank the reviewer for these detailed insights. Yes, perhaps there could be some spurious or omitted results. Our main goal here was to unlock DisGeNET, a published and well cited resource, to command-line usage for clinical diagnostic purposes. To find out how the resulting tool would behave, we constructed a benchmark using 305 real patient cases from a completely independent clinic. From the results, we indeed discovered a form of bias that yet remains to be solved as future work. We added this to the "Discussion" (p.4) section and have created a Github issue for this problem at https://github.com/molgenis/vibe/issues/35. We are fully commited to continue improving VIBE further by solving this issue as well as other problems that may come to our attention. In the version 2.0, already many things were improved compared to version 1.0, and version 3.0 is planned.

Comment: Seventh, the candidate diagnoses come seemingly from OMIM and MeSH. It is well known

that there are many Mendelian diseases not in MeSH, that the MeSH disease hierarchy is not an adequate computational representation, and there are a number of additional sources of disease information that may provide different results. It is not clear if additional disease-gene sources are included within DisGeneNet, are used for the prioritization, but then not reported in the candidate results? Why would DisGeneNet use MeSH for example to reveal disease pleiotropy? I would be very careful with such a measure and base it on a more robust disease terminology and its associations (or perhaps I misunderstood).

Response: This is a valid point. First, we have now clarified which sources VIBE uses to map phenotypes to diseases (and subsequently genes) in the “Implementation” (p.2) and “Algorithm” (p.2) sections. These are: HPO to CUI matching according to UMLS Metathesaurus, ORDO matching according to HOOM and other associations according to DisGeNET PDA. Second, we agree that disease pleiotropy (DPI) was not a good measure. In fact, testing revealed that GDA_Max works better than both DSI and DPI under every circumstance. To make things faster and easier, we removed DSI and DPI from the tool implementation and cmdline options in version 2.0 (the version presented in the manuscript).

Comment: Eighth, this reviewer concurs regarding the use of current gene nomenclature and with the frustrations that come from this issue. However, any good pipeline should be able to track provenance of gene nomenclature in data sources, flag issues, and convert to current nomenclature and identifiers using a variety of APIs and services, for example MyGene.info.

Response: We fully agree that nomenclature issues should have be resolved. We have rectified this by converting all tool output genes to NCBI gene identifiers, and now have significantly fewer missed genes in the benchmark, showing that this indeed needed to be done. In fact, VIBE v2.0 now outputs NCBI gene identifiers to prevent such issues in the future. Since this issue no longer applies, we have removed it from the “Discussion” (p.4) section.

Comment: In summary, VIBE poses promising use of the integrated literature and knowledge within DisGeneNet in the context of variant prioritization, but perhaps the focus should be on its incorporation into existing pipelines and tools as a corroboration feature rather than a standalone too. Most clinical pipelines leverage a multiplicity of algorithms and methods and the best methods for integration of DisGeneNet might be the win here.

Response: We thank the reviewer for this suggestion. We agree that VIBE needs to be incorporated into genomic analysis/interpretation pipelines to show its real value. We are already thinking of projects to combine VIBE with complementary gene prioritizers, state-of-the-art variant prioritizers (e.g. CAPICE), and others, into a novel composable pipeline for NGS genome diagnostics and research, and publish if that would lead to advances or insights in this field. The VIBE manuscript and its benchmarks would be one of the foundations for such future work.

2nd Editorial Decision	March 20, 2020

There are some minor edits in the manuscript (see the attached manuscript), please make a change accordingly, if acceptable. Our journal does not allow a colon in the title, so I included an edited version for you to consider making a change.

It was a great pleasure to work with you. Thank you for contributing to the GGN. Please continue to consider us in your future submission.

Alison Liu, Ph.D.
Editor, Genetics & Genomics Next

2nd Review	March 20, 2020

Reviewer: 1

Comments to the Author: The authors have now addressed the key issue raised by all 3 reviews, namely using relative ranks, as well as my minor comments.

Reviewer: 3

Comments to the Author: The manuscript has significantly improved after revision. I have no additional comments.

Reviewer: 4

Comments to the Author: The authors have addressed all of my concerns in this revised version.
